# On the Prognostic Power of Tumor-Infiltrating Lymphocytes – A Critical Commentary

**DOI:** 10.3389/fimmu.2022.892543

**Published:** 2022-05-12

**Authors:** Zeev Elkoshi

**Affiliations:** Research and Development Department, Taro Pharmaceutical Industries Ltd, Haifa, Israel

**Keywords:** tumor-infiltrating lymphocytes, regulatory T cells, CD8+ T cells, cancer prognosis, CD8/Treg, Treg

## Abstract

Tumor-infiltrating lymphocytes are extensively used as prognostic biomarkers in cancer. Regulatory T cells (Tregs) or CD8+ T cells frequencies in tumor site, or their ratio, are the most common markers used to assess prognosis. This work offers a possible explanation for the opposite correlations between intra-tumoral Tregs and survival, associated with different types of cancer. The complexity involved with the selection of a preferred marker, including the effect of variability, is presented and discussed. The lymphocytes frequency ratio is proposed as the marker of choice in most types of cancer. The ratio correlates directly with survival, irrespective of cancer type and is also less variable than the frequencies of each of the two lymphocytes, if these frequencies correlate with each other in the tumor microenvironment. However, if the frequency of one of the two lymphocytes is highly variable, abandoning the ratio in favor of the lymphocyte with less variable frequency will improve correlation with survival, especially when the intra-tumoral frequencies of the two species are inversely correlated. It is plausible, that the best prognostic marker selected this way, will be also be the best predictor of checkpoint inhibitor therapy success.

## Introduction

Tumor-infiltrating lymphocytes can affect cancer progression. Tumor-infiltrating lymphocytes may generally be classified as tumor-suppressive or tumor-promoting lymphocytes.

Cytotoxic CD8+ T cells play a major role in sustaining anti-cancer immunity by attacking cancer cells directly (through FAS-mediated apoptosis and perforin-mediated cytolysis) (1). Within the tumor microenvironment (TME), regulatory T cells (Tregs) are the major tumor-suppressive lymphocytes (2). Regulatory T cells suppress the anti-cancer activities of CD8+ T cells and of CD4+ T cells and dendritic cells (DCs) that mediate CD8+ T cell activation. Diverse contact-dependent and cytokine-mediated mechanisms are employed by Tregs for this purpose, as thoroughly reviewed by Han et al. (3). For example, perforin and granzyme expressed by Tregs in the TME (but not by naïve Tregs) trigger lysis of effector T cells (and of NK cells) (4). In addition, CD39 and CD73 expressed on Treg cells catalyze adenosine generation which suppresses the anti-tumor function of other T cells (5). It is not surprising therefore, that CD8+ and regulatory T cells are vastly used as markers for cancer prognosis. This commentary provides a possible explanation for the opposite correlations between intra-tumoral Tregs and survival, affiliated with different types of cancer. It also proposes simple rules for the selection of preferred prognostic biomarkers, considering the variability in frequency and function of intra-tumoral lymphocytes.

## Intra-Tumor Accumulation of Tregs May Correlate With a Better or Worse Prognosis, Depending on Cancer Type. A Possible Explanation

Shang et al. performed a meta-analysis to assess the prognostic value of Tregs (FoxP3+ T cells) across different types of cancer (6). Seventeen types of cancer and 15,512 cancer cases were analyzed. Using intra-tumoral Tregs as a marker, and a 95% confidence interval, cervical cancer, lung cancer, renal cancer, ovarian cancer, hepatocellular carcinoma, melanoma, and pancreatic cancer, positively correlated with a shorter survival while colorectal cancer, head and neck cancer, endometrial cancer, and esophageal cancer correlated with a longer survival compared to cancer-specific mean values.

The present work proposes an explanation for the direct or inverse correlations of intra-tumoral Tregs with survival, observed in different cancer types. The clue for this puzzle lies in the opposite effects of CD8+ T cells and Tregs on cancer growth, coupled with different penetration rates of the two lymphocytes into the TME. In some types of cancer, impairment of CD8+ T cell anti-cancer activity in the TME also affects the relation between Tregs accumulation in tumor and cancer prognosis.

Suppose that intra-tumoral cell frequencies of CD8+ T cells and Tregs correlate directly with each other. Suppose that Treg frequency is used as a marker. Any increase in intra-tumoral Treg cell number will be associated with an increase in CD8+ cell number. Since CD8+ T cells have a positive effect on survival, prognosis will be better than expected if the two frequencies were independent of each other. Accordingly, the hazard ratio (HR) will be smaller than expected if correlation between the frequencies is not assumed.

Consider the opposite scenario, when intra-tumor frequencies of the two lymphocytes inversely correlate with each other. Suppose that Treg frequency is used as a marker. Any increase in intra-tumoral Treg cell number will be associated with a decrease in CD8+ cell number. Since CD8+ T cells have a positive effect on survival, prognosis will be worse than expected if the two lymphocytes were independent of each other. The hazard ratio (HR) in this case will be larger than expected if correlation between the frequencies is not assumed.

Inspecting published experimental data, it seems that the effect of intra-tumoral Tregs on CD8+ tumor infiltration depends on the specific type of cancer. As will be demonstrated below, in cancer types presenting HR < 1, an increased intra-tumoral Tregs is involved with an increased infiltration of CD8+ T cells. On the other hand, in many cancer types presenting HR>1, an increased intra-tumoral Tregs is involved with a decreased infiltration of CD8+ T. In line with this, in several types of cancer with HR >1, CD8+/Treg frequency ratio is reduced at higher tumor grades. In addition, in several cancer types with HR > 1, an impaired cytotoxic function of CD8+ T cells has been reported. Each of these properties contributes to the effect of intra-tumoral Tregs on survival. The examples below illustrate these points.

### Hazard Ratio < 1

#### Colorectal Cancer (CRC)

Sideras et al. reported a positive (though not statistically significant) effect of tumor penetration by FoxP3 cells on survival of CRC patients with liver metastases, after metastatectomy. At the same time, the intra-tumoral CD8/FoxP3 cell ratio was an independent positive predictor of survival (7). Similar results were observed by Suzuki et al. (8). Assuming a pro-cancer role for Tregs and an anti-cancer role for CD8+ T cells, such an event is possible only if the two lymphocytes infiltrate simultaneously into the tumor, where the positive effect of CD8+ T cells overweighs the negative effect of FoxP3+ T cells on cancer prognosis. In accordance with this, several works demonstrated a positive correlation between the frequencies of intra-tumoral CD8+ T cell and Tregs in colorectal cancer (9–12). In addition, CD8+ T cell densities at high tumor stages were similar to these at lower tumor stages (the difference was statistically non-significant), while Treg cell densities at high tumor stages were statistically significantly lower compared to lower stages (T_3+4_ vs. T_1+2_; P = 0.007) (11). This behavior however did not repeat using the AJCC staging system. At AJCC stage III, both CD8+ and Treg densities were reduced compared to their values at AJCC stage II, but the reduction in CD8+ T cell density was larger (11).

#### Head and Neck Cancer

Echarti et al. compared lymphocyte densities in head and neck tumor tissue samples with different degrees of lymphocyte infiltration (13). The authors noticed that CD8+ and FoxP3+ T cells infiltration occurred simultaneously. However, the CD8+/FoxP3 frequency ratio was higher in tumor epithelia than in stroma indicating a higher influx of CD8+ cells than Tregs into tumor epithelia. This difference in flow rate may contribute to the beneficial effect of intra-tumoral Tregs on the prognosis of head and neck carcinoma.

#### Hypopharyngeal Squamous Cell Carcinoma (HSCC)

Wang et al. noticed a beneficial effect of both tumor-infiltrating Tregs and of the CD8/FoxP3 density ratio on the survival of HSCC patients (14).The simultaneous validity of these two observations can hold only if the positive effect of CD8+ T cells overweighs the negative effect of Tregs on prognosis, considering the opposite effects of the two lymphocytes on tumor growth.

#### Ovarian Cancer (Advanced Stage)

In advanced stage ovarian cancer, the presence of CD8+ cells, FoxP3+Treg or a high CD8/FoxP3 frequency ratio in tumor tissue was associated with an increased disease specific survival (15). Similarly, disease-specific survival was positively associated with the markers CD8 and FoxP3 in high-grade serous tumors from optimally debulked patients (16). These scenarios can hold only if the beneficial effect of tumor-infiltrated CD8+ T cells on patients survival, more than compensate for the detrimental effect of tumor-infiltrating Tregs on survival.

### Hazard Ratio > 1

#### Lung Cancer

An inverse correlation between CD8+ T cells and Tregs intra-tumor frequencies was reported in lung adenocarcinoma tumors, whereby CD8+ T cell frequency reduced while Tregs frequency increased at the tumor site compared to non-involved lung tissue (17). Jackute et al. reported an increase in both CD8+ cells and Tregs numbers in non-small cell lung cancer tumors, in comparison to controls, but the increase in Treg cells number was double the increase in the number of CD8+ T cells (18). In a mouse model of pulmonary adenocarcinoma, Tregs accumulated over the time in tumor tissues and induced tumor growth, while CD8+ T cells restrained this growth (19). In addition, Tregs were elevated in the circulation of patients with untreated extensive stage small cell lung cancer, compared to healthy controls. These circulating Tregs negatively correlated with the percentage of proliferative CD8+ T cells in peripheral blood (20).

#### Renal Cell Carcinoma (RCC)

Using specimens collected from RCC patients, Kawashima et al. performed RNA sequencing of both CD8+ T cells and CD4+ T cells based on the expression patterns of PD-1 and TIM-3 in tumor and adjacent normal tissue. Among these T cells, a sub-population of regulatory CD4+ T cells (Tregs) and a subpopulation of *exhausted* CD8+ T cellswere identified. These two subpopulations accumulated more in high-grade RCC tumors than in low-grade tumors (21). It turns out that RCC high-grade tumors are infiltrated more by Tregs and less by active (unexhausted) CD8+ T cells than low-grade tumors.

#### Endometrial Cancer

The infiltration of CD8+ T cells and Tregs into endometrial cancer tumors was evaluated by Yamagami et al. using immunohistochemistry. Both CD8+ and Treg cell counts as well as the Treg/CD8+ count ratio increased with higher tumor grades, implying a larger increase in Treg frequency compared to CD8+ frequency at higher tumor grades. Disease free survival (DFS) was shorter in patients with high Treg counts or a high Treg/CD8 count ratio (22) [in opposite to the pooled analysis by Shang et al. (6)]. In addition, it was reported that endometrial cancer cells suppressed CD8+ T cell cytotoxicity (23). This effect counteracts the effect of the increased number of CD8+ T cells and further explains the association between high tumor infiltration by Treg cells and shorter survival of endometrial cancer patients.

#### Cervical Cancer

In a study including 115 cervical cancer patients, the mean frequencies of both, CD8+ T cells and Tregs, increased considerably in tumor tissue compared to cervical tissues excised from women with no cervical abnormalities. However, the increase in Tregs frequency was double the increase in CD8+ frequency. In this study, both, low FoxP3 frequency and high CD8+/FoxP3 frequency ratio in tumor tissue correlated with a longer survival (24). A study by Shah et al. confirmed the negative effect of high intra-tumoral Tregs on the survival of cervical cancer patients (25). The higher influx of Tregs into tumor tissue compared to that of CD8+ cells may explain the negative correlation between high intra-tumoral Tregs and survival in cervical cancer.

#### Ovarian Cancer

Adams et al. found that high frequency of intra-tumoral FoxP3+ T cells in ovarian adenocarcinoma specimens was statistically significantly associated with diminished long-term survival. They noticed that patients who had tumors with a high frequency of intraepithelial CD8+ cells and a low frequency of FoxP3+ T cells had a 5-year survival rate of 64.3%, while patients with high frequency of intraepithelial CD8+ T cells and high frequency of FoxP3+ T cells had a much lower survival rate of 32.1% (26). It is clear that here, the negative effect of intra-tumoral Tregs on patients survival, overcomes the positive effect of CD8+ T cells. As aforementioned, in *advanced* ovarian cancer, intra-tumoral high Tregs frequency is associated with *improved* survival. A change in the relative tumor penetration rates of CD8+ T cells and Tregs, between advanced and early cancers, may be the cause for this discrepancy. Such a change, however, is not documented in the literature.

#### Hepatocellular Carcinoma (HCC)

In a pooled analysis of HCC studies, high intra-tumoral infiltration of Tregs was associated with a mean HR value of 1.894 (95% CI: 1.658 – 2.164) for overall survival, while high infiltration of CD8+ T cells was associated with a mean HR value of 0.676 (95% CI: 0.540 – 0.845) for overall survival (27). An increased frequency of Tregs together with a decreased frequency of CD8+ T cells was observed in HCC tumor regions (relative to healthy tissue) (28, 29). In addition, higher histologic grades of HCC tumors were associated with a higher FoxP3/CD8 frequency ratio (30). An impairment of intra-tumoral CD8+ cell cytotoxic function by HCC intra-tumoral Tregs was also reported (28).The association between high infiltration of Tregs on the one hand, and low infiltration of CD8+ cells on the other hand, coupled with a decreased activity of intra-tumoral CD8+ cells, contribute to the positive correlation between high intra-tumoral Tregs and poor survival in HCC.

#### Pancreatic Cancer

Two meta-analyses have shown a high negative impact of intra-tumoral Tregs on the prognosis of pancreatic cancer (6, 31). The relative numbers of intra-tumoral CD4+CD25+Foxp3+ Tregs and CD8+ T cells were negatively correlated in pancreatic ductal adenocarcinoma (32).

These results are summarized in **Table 1**.

**Table 1:** Hazard ratios associated with Tregs infiltration and the related lymphocyte frequencies in the TME.

Cancer Type	HR associated with high tumor infiltration by Tregs	Reference	Intra-tumoral CD8+ T cells	Intra-tumoral Treg cells	References
Colorectal cancer	HR < 1	(6)^(^*^)^	CD8+ and Treg tumor frequencies are positively correlated. CD8+ effect overweighs Tregs effect.	CD8+ and Treg tumor frequenciesare positively correlated. CD8+ effect overweighs Tregs effect.	(7–12)
Colorectal cancer	HR < 1	(6)^(^*^)^	Intra-tumoral CD8+ density is independent of tumor stage	Intra-tumoral Treg density is lower at higher tumor stages	(11)
Head and neck cancer	HR < 1	(6)^(^*^)^	Tumor frequencies of CD8+ and Tregs are positively correlated. CD8+ effect overweighs the effect of Tregs.CD8+/Treg frequency ratio is higher in tumor epithelia than in stroma	Tumor frequencies of CD8+ and Tregs are positively correlated. CD8+ effect overweighs the effect of Tregs. CD8+/Treg frequency ratio is higher in tumor epithelia than in stroma	(13, 14)
Ovarian cancer (advanced stage)	HR < 1	(15, 16)	Both CD8+ and Tregs infiltrate into tumor simultaneously. CD8+ effect overweighsTregs effect	CD8+ and Tregs infiltrate into tumor simultaneously. CD8+ effect overweighs Tregs effect	(15, 16)
Ovarian cancer	HR > 1	(26)	Intra-tumoral Tregs effect overweighs intra-tumoral CD8+ effect	Intra-tumoral Tregs effect overweighs intra-tumoral CD8+ effect	(26)
Lung cancer	HR > 1	(6)^(^*^)^	Intra-tumoral CD8+ frequency is lower relative to normal tissue	Intra-tumoral Treg frequency is higher relative to normal tissue	(17)
Lung cancer	HR > 1	(6)^(^*^)^	Intra-tumor Tregs number is higher than CD8+ number (both are higher compared to normal tissue)	Intra-tumor Treg number is higher than CD8+ number (both are higher compared to normal tissue)	(18)
Lung cancer	HR > 1	(6)^(^*^)^	Percentage of proliferative *circulating* CD8+ inversely correlate with *circulating* Tregs	*Circulating* Tregs elevated and inversely correlate with percentage of proliferative *circulating* CD8+	(20)
Renal cell carcinoma	HR > 1	(6)^(^*^)^	Active CD8+ frequency is lower in high-grade tumors	Treg frequency is higher in high-grade tumors	(21)
Endometrial cancer	HR > 1	(22)	CD8+ frequency increases but less than Treg frequency at higher tumor grades. Endometrial cancer cells suppress CD8+ activity	Treg frequency increases more than CD8+ frequency at higher tumor grades	(22, 23)
Cervical cancer	HR > 1	(24, 25)	Both CD8+ and Treg frequencies increase in tumor compared to normal tissue. CD8+ increase is half the increase in Tregs frequency	Both CD8+ and Treg frequencies increase in tumor compared to normal tissue.Treg frequency increase is double the increase in CD8+ frequency	(24)
Hepatocellular carcinoma	HR > 1	(27)^(^*^)^	CD8+ frequency in tumor is reduced and CD8+ function is impaired. CD8+/Treg frequency ratio is lower at higher tumor grades	Tregs frequency in tumor is increased. CD8+/Treg frequency ratio is lower at higher tumor grades	(28–30)
Pancreatic cancer	HR > 1	(6)^(^*^)^ (31)^(^*^)^	Intra-tumoral CD8+ and Treg numbers were negatively correlated	Intra-tumoral CD8+ and Treg numbers were negatively correlated	(32)

HR, hazard ratio (mortality hazard associated with Tregs infiltration into the TME, compared to cancer-specific mean values).

(*)The reference presents mean values obtained by a pooled analysis of the variables.

## Tumor-Infiltrating CD8+ T Cells, Treg Cells or CD8+/Treg Ratio: Which Is the Preferred Prognostic Marker?

Selecting the best prognostic marker is important not only for improving cancer prognosis. A better prognostic marker may better correlate with the response to checkpoint inhibitor therapy.

By the earlier discussion, it is clear that survival is a function of both intra-tumoral lymphocytes, Tregs and CD8+ cells. However, due to the opposite effects of these two lymphocytes on survival, it seems that the frequency ratio would better correlate with survival, than the frequency of each of the single species. In addition, the use of the CD8/Treg frequency ratio as a marker is expected to correlate directly with cancer prognosis, *irrespective* of cancer type or stage, unlike a prognosis based on a the frequency of CD8+ cells or Tregs.

However, more confounding factors are involved in the process of selecting the “the best” marker. In particular, it should be realized that the addition of any extra variable may affect variability. In fact, when x and y are two correlated random variables, the variance (σ) of {x/y}can be approximated by (33):


(1) $$\text{σ}(\text{x}/\text{y})∼\left(\mu_{\text{x}}^{2}/\mu_{\text{y}}^{2}\right)\left\{\sigma_{\text{x}}^{2}/\mu_{\text{x}}^{2}-2\text{Cov}(\text{x},\text{y})/\mu_{\text{x}}\mu_{\text{y}}+\sigma_{\text{y}}^{2}/\mu_{\text{y}}^{2}\right\}$$

Where: σ = variance; Cov = covariance; µ = arithmetic mean;

By eq. 1, when x and y directly correlate with each other (Cov> 0), the variance of {x/y} is lower compared to the variance involved with independent (uncorrelated) variables (Cov=0), assuming fixed µ_x_, µ_y_, σ_x_, σ_y_ values. Under the same assumption, when x and y are inversely correlated (Cov< 0), the variance of {x/y}is higher compared to the variance involved with independent variables.

Thus, the prognostic power of the ratio is inherently poorer when CD8+ cell and Treg frequencies are inversely correlated, compared to a situation when they are directly correlated, assuming all other variables in eq. 1 are fixed.

When HR decreases with a higher Tregs intra-tumoral frequency, the two lymphocytes intra-tumoral frequencies necessarily correlate directly with each other (Cov>1). Both lymphocytes infiltrate simultaneously into the tumor tissue, but CD8+ T cells infiltrate faster than Tregs, resulting in improved prognosis. **Table 1** includes 3 types of cancer with HR < 1. Indeed, in CRC (9–12), in head and neck cancer (13, 14), and in advanced ovarian cancer (15, 16), the tumoral frequencies of the two species positively correlate with each other (comparing tumors of different grades or comparing tumor with normal tissue).

When HR increases with higher frequencies of intra-tumoral Tregs, the two lymphocyte frequencies either inversely correlate with each other (Tregs frequency increases while CD8+ cells frequency decreases) (Cov<1), or they are directly correlated(Cov>1), however in the last scenario Tregs are expected to infiltrate faster than CD8+ T cells into the tumor site, affecting an increase in HR this way. **Table 1** includes 7 types of cancer with HR > 1. In lung adenocarcinoma (17), renal cell carcinoma (21), HCC (28, 29), and pancreatic cancer (32), the frequencies of Tregs and CD8+ (or active CD8+) are inversely correlated. In ovarian cancer (26), and cervical cancer (24) these frequencies are directly correlated. In endometrial cancer, even though the frequencies directly correlate with each other (22), the tumor microenvironment downregulates the activity of CD8+ T cell (23), and the number of *active* CD8+ T cells plausibly decreases.

Consequently, for most types of cancer listed in Tab. 1, when intra-tumoral *Tregs* frequency is used as a marker for cancer prognosis, equation 2 holds:


(2) $$\left\{{\text{σ}}_{\text{HR}},\text{when HR}>1\}>\{{\text{σ}}_{\text{HR}},\text{when HR}<1\right\}\;$$

Where HR is the mortality hazard ratio affected by an increase in intra-tumoral Tregs frequency.

Inspection of the data presented in Shang et al. meta-analysis (6) (which is based on Tregs as a marker) confirms eq. 2 for *all* cancer types: within this list of 11 types of cancers, any cancer type with HR > 1, presents a larger variance than any cancer type with HR < 1.It should be realized that the hazard involved with an increase in Tregs frequency is different from the hazard involved with an increase in the lymphocytes frequency ratio. Thus, eq. 2 does not hold when the lymphocytes frequency ratio is used as a marker.

As mentioned above, dendritic cells and effector CD4+ T cells also affect survival, since both types of cells are involved in priming cytotoxic reaction (34). In addition, effector CD4+ T cells may demonstrate direct cytotoxicity (35).The control of dendritic cells and CD4+T effector cells by regulatory T cells adds to the complexity involved with the prediction of prognosis.

Due to these intricate relationships it seems that no single marker would fit all types of cancer and all patient subpopulations as “the best” prognostic marker.

Having said that, the frequency ratio of the two lymphocytes(CD8+/Treg or Treg/CD8+) demonstrates good correlation with survival in many types of cancer. A good correlation of the ratio with survival was observed in lung adenocarcinoma (17), cervical cancer (24), type I endometrial cancer (36), ovarian cancer (37, 38), colorectal cancer (7, 8), endometrial cancer (overall survival) (22), and breast cancer (39). In some of these studies (7, 8, 24, 37, 38) the frequency ratio was found superior to the single lymphocyte frequencies (one of them or both) as a prognostic marker. Moreover, {PD1+Tregs/PD1+CD8+T cells} ratio was found superior to all other markers in predicting the efficacy of programmed cell death protein 1 (PD-1) blockade therapies (40).

However, the frequency ratio did not correlate with survival in three clinical studies: in type II endometrial cancer study (36), in a CRC study (41), and in endometrial cancer study (DFS) (22). In addition, a meta-analysis of 21 ovarian cancer studies did not show a correlation of the ratio with survival (42).

Taken together, the use of the lymphocytes frequency ratio as a marker is recommended when the frequency variances of the two lymphocytes within the tumor site are of comparable size, or when their values are unknown. However, if one lymphocyte presents highly variable frequency or function, the other specie should be preferred as a marker. In such a case, the lymphocyte frequency ratio may demonstrate high variability and may poorly correlate with survival, especially when the intra-tumoral frequencies of the two species inversely correlate with each other.

## Summary

This commentary provides a possible explanation for the opposite correlations between intra-tumoral Tregs and survival, associated with different types of cancer. For most types of cancer, it also explains the higher variances of hazard ratios (σ_HR_) observed in cancer types with HR>1 compared to cancer types with HR<1, when Tregs frequency in the tumor microenvironment is used as a marker for cancer prognosis. The complexity involved with the selection of a preferred prognostic marker is presented and discussed. The lymphocytes frequency ratio is proposed as the marker of choice, in the absence of data regarding the variances of the two lymphocytes (frequency or function) within the tumor microenvironment, or if the two variances are of comparable size. If the intra-tumoral frequency of one of these two species exhibits high variability, the low variable lymphocyte should be preferred, over both, the highly variable lymphocyte and the frequency ratio, especially when the intra-tumoral frequencies of the two lymphocytes inversely correlate with each other. The best prognostic marker selected this way, may also be the best predictor for checkpoint inhibitor therapy of cancer. **Table 2** summarizes these results.

**Table 2:** A summary table of (a) mortality hazard ratios associated with the use of different intra-tumoral markers; (b) the preferred markers under different relationships between lymphocyte-related variances.

	Tregs	CD8+/Tregs	CD8+
frequencies inversely correlate with each other	HR > 1	HR < 1	HR < 1
frequencies directly correlate with each other {TIR(CD8) >TIR(Treg)}	HR < 1	HR < 1	HR < 1
frequencies directly correlate with each other {TIR(CD8) <TIR(Treg)}	HR > 1	HR < 1	HR > 1
σ_CD8_ and σ_Treg_ are of comparable size, or are unknown	_	preferred	_
σ_CD8_>>σ_Treg_	preferred	_	_
σ_CD8_<<σ_Treg_	_	_	preferred

TIR(X), tumor infiltration rate (X); σ_x_, intra-tumoral variance of specie X; HR, hazard ratio associated with an increase in the marker’s value.

## Author Contributions

The author confirms being the sole contributor of this work and has approved it for publication.

## Author Disclaimer

The views and opinions expressed, and/or conclusions drawn, in this article are those of the author and do not necessarily reflect those of Taro Pharmaceutical Industries Ltd., its affiliates, directors or employees.

## Conflict of Interest

The author is an employee of Taro Pharmaceutics, Israel.

## Publisher’s Note

All claims expressed in this article are solely those of the authors and do not necessarily represent those of their affiliated organizations, or those of the publisher, the editors and the reviewers. Any product that may be evaluated in this article, or claim that may be made by its manufacturer, is not guaranteed or endorsed by the publisher.
